# Differentiation-Induced Remodelling of Store-Operated Calcium Entry Is Independent of Neuronal or Glial Phenotype but Modulated by Cellular Context

**DOI:** 10.1007/s12035-018-1112-y

**Published:** 2018-05-26

**Authors:** Claire L. Whitworth, Christopher P. F. Redfern, Timothy R. Cheek

**Affiliations:** 1 Institute for Cell and Molecular Biosciences, Medical School, Newcastle University, Newcastle upon Tyne, NE2 4HH UK; 2 Present Address: Division of Biological Chemistry & Drug Discovery, School of Life Sciences, University of Dundee, Dundee, Scotland DD1 5EH UK; 3 Northern Institute for Cancer Research, Medical School, Newcastle University, Newcastle upon Tyne, NE2 4HH UK

**Keywords:** Neurogenesis, Retinoic acid, Differentiation, calcium signalling, Orai1, STIM1, Neuronal, Glial, Neuroblastoma, Store-operated calcium entry

## Abstract

Neurogenesis is a complex process leading to the generation of neuronal networks and glial cell types from stem cells or intermediate progenitors. Mapping subcellular and molecular changes accompanying the switch from proliferation to differentiation is vital for developing therapeutic targets for neurological diseases. Neuronal (N-type) and glial (S-type) phenotypes within the SH-SY5Y neuroblastoma cell line have distinct differentiation responses to 9-*cis*-retinoic acid (9*c*RA). In both cell phenotypes, these were accompanied at the single cell level by an uncoupling of Ca^2+^ store release from store-operated Ca^2+^ entry (SOCE), mediated by changes in the expression of calcium release-activated calcium pore proteins. This remodelling of calcium signalling was moderated by the predominant cell phenotype within the population. N- and S-type cells differed markedly in their phenotypic stability after withdrawal of the differentiation inducer, with the phenotypic stability of S-type cells, both morphologically and with respect to SOCE properties, in marked contrast to the lability of the N-type phenotype. Furthermore, the SOCE response of I-type cells, a presumed precursor to both N- and S-type cells, varied markedly in different cell environments. These results demonstrate the unique biology of neuronal and glial derivatives of common precursors and suggest that direct or indirect interactions between cell types are vital components of neurogenesis that need to be considered in experimental models.

## Introduction

Mapping subcellular and molecular changes that accompany the cellular switch from proliferation to differentiation is vital for developing therapeutic targets for neurological diseases. Neurogenesis in the central and peripheral nervous systems is a complex process, involving the generation of diverse networks of neuronal cells and their supporting glial cell types from neuronal stem cells or from intermediate progenitor cells (IPCs; [1]). Many studies of neuronal differentiation in vitro have used human neuroblastoma cell lines as models of human disease, and the SH-SY5Y cell line has attracted particular use as a model for Parkinson’s disease [2]. Whilst it is possible, with special manipulation, to obtain homogenous populations of neuronal SH-SY5Y cells [3], this cell line is of particular interest because both neuronal and glial-like cells can be identified within SH-SY5Y cultures. The cell line represents a powerful approach for studying the processes of neuronal and glial lineage development from the putative IPCs or I-type SH-SY5Y cells which can be identified within SH-SY5Y cell populations.

**Fig. 1:**
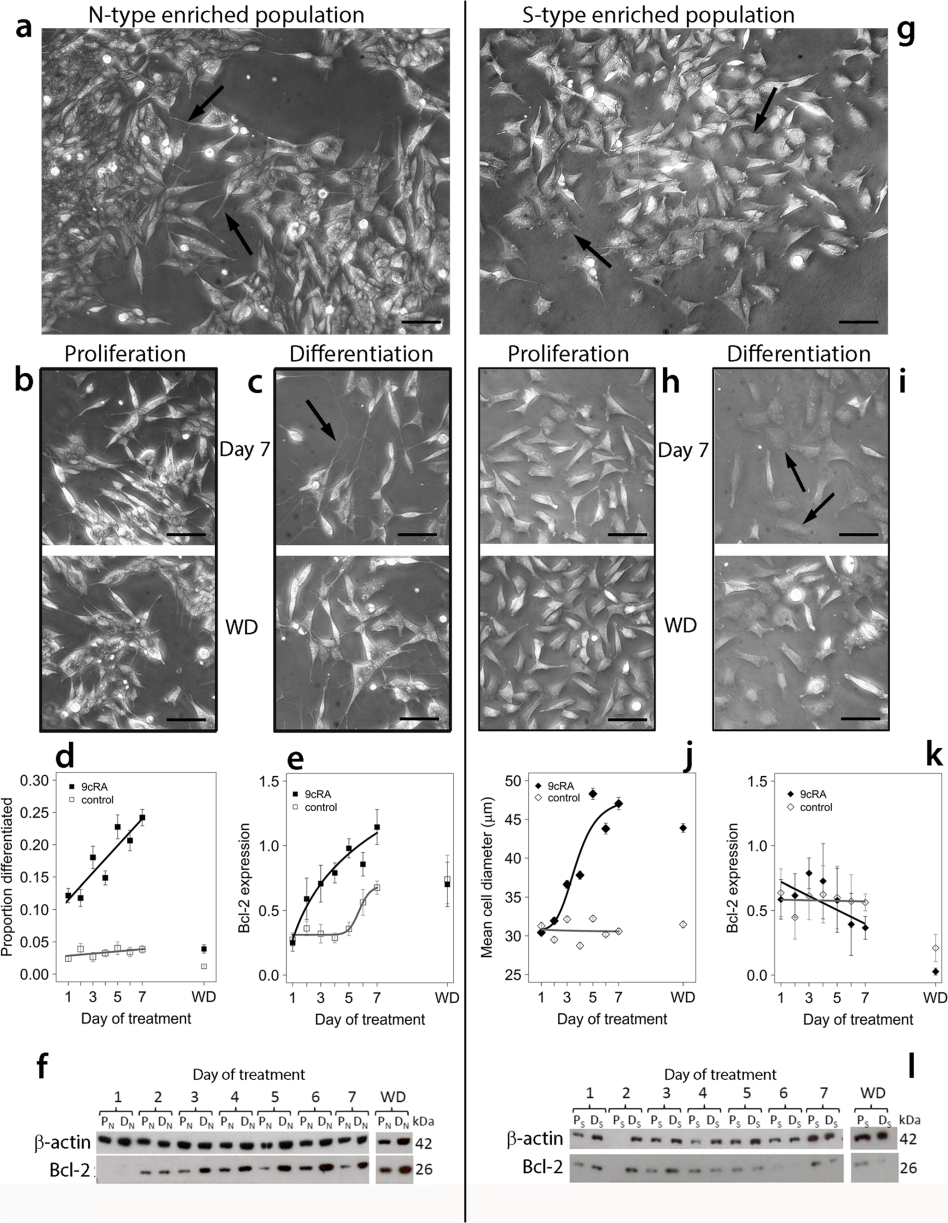
Differentiation of N-type-enriched and S-type-enriched populations. N-type (**a**) or S-type enriched populations (**g**) were treated for up to 7 days with 1 μM 9*c*RA to induce differentiation (**c**, **i**) or an equivalent volume of EtOH for proliferating controls (**b**, **h**) after which treatment was withdrawn and cells were grown for a further 5 days (WD). Scale bars (phase-contrast images) represent 50 μm. Cells proliferating within N-type-enriched populations had short, neurite-like processes (**a**, arrows) and often grew in aggregates. Differentiating N-type-enriched populations contained cells with elongated neurites (**c**, day 7), whereas after washout cultures they were similar to proliferating cultures (**b**, **c**, WD). Within proliferating S-type-enriched populations, cells had abundant cytoplasm and a flattened morphology (**g**, arrows). Cells within differentiating S-type populations frequently exhibited a flattened morphology with spread cell margins (**i**, day 7; arrows), retained after washout (**i**, WD). Over the treatment time course, there was a significant increase in proportion of differentiated cells in N-type-enriched populations treated with 9*c*RA (**d**, 488–1192 cells per time point) compared to proliferating populations (**d**, 398–1084 cells per time point; linear model, effect of time *F*_1,10_ = 24.3, *P* < 0.001; differentiation *F*_1,10_ = 247.6, *P* < 0.0001; interaction *F*_1,10_ = 17.6, *P* < 0.002). After washout (WD), the proportion of differentiated cells returned to baseline (**d**). For cells in S-enriched populations, there was a significant effect of 9*c*RA (**j**, 399–543 cells per time point) in increasing cell diameter compared to proliferating cells (**j**, 315–678 cells per time point; differentiation effect, *F*_4,7171_ = 468.8, *P* < 0.001) which remained after washout. In N-type-enriched cultures, Bcl-2 expression (normalised to β-actin) increased after treatment with 9*c*RA (**e**, example blot in **f**), but increased to a lesser extent and only after 6–7 days in proliferating cultures (**e**; differentiation effect, linear mixed-effect analysis: *P* < 0.001). After washout, Bcl-2 expression was similar in differentiating and proliferating cultures (**e**; **f**: example blot of a single experiment [filter was torn at day 5 P_N_ track during processing and image re-joined]). For S-type-enriched populations, there was a small but significant difference in Bcl-2 expression between proliferating and differentiating populations (**k**; example blot in **l**) over the treatment time course (effect of differentiation: *P* = 0.05), and a significant difference between proliferating and differentiating cells over time (interaction: *P* = 0.004). All error bars are ± SEM

At least three distinct phenotypes can be recognised in the SH-SY5Y cell line. N-type cells (Fig. 1a) are immature neuronal precursors which express neuronal cell markers such as neurofilament protein [4], dopamine β-hydroxylase [4, 5] and β-tubulin III [6]. N-type cells differentiate towards a neuronal lineage in response to compounds such as 9*-cis*-retinoic acid (9*c*RA) [6]. Substrate adherent or glial-like S-type cells (Fig. 1g) express vimentin [6], fibronectin and tyrosinase [4, 7], markers characteristic of non-neuronal stromal or glial cells. S-type cells respond to 9*c*RA by becoming more epithelial-like with a flattened morphology [6]. I-type cells exhibit characteristics of both N- and S-type cells and may represent a multi-potent progenitor population [4, 7]. Phenotypic interconversion can occur between N-type and S-type cells [4, 5].

Intracellular free Ca^2+^ is a ubiquitous signalling ion, regulated by release from endoplasmic reticulum (ER) stores and uptake from the cellular milieu, and plays a vital role in cell differentiation [8–11]. Store-operated Ca^2+^ entry (SOCE) is mediated by STIM1, a Ca^2+^-binding protein that senses endoplasmic reticulum (ER) Ca^2+^ store depletion [12–14], and Orai1, which forms the pore of Ca^2+^ release-activated Ca^2+^ (CRAC) channels [12–17]. STIM1 oligomerises and forms puncta with the plasma membrane (PM) after ER Ca^2+^ store depletion and directly gates Orai1 to trigger cytosolic influx of extracellular Ca^2+^ [12, 15, 18]. This process is crucial for replenishing depleted intracellular ER Ca^2+^ stores and prolonging the Ca^2+^ response for downstream signalling [9, 19–21]. We have previously shown that 9*c*RA-induced differentiation of SH-SY5Y neuroblastoma cell populations induces an uncoupling of SOCE from ER Ca^2+^ store release [6, 22] coinciding with STIM1 and Orai1 down-regulation [6]. Although evidence suggested that S-type SH-SY5Y cells do not show an uncoupling of these two processes [6], this has not been investigated at the single-cell level. This study tests the hypothesis that N- and S-type cells differ in their differentiation response with respect to Ca^2+^ release and SOCE, and investigates the cellular context of such responses with respect to potential modulating effects of different cell phenotypes.

## Material and Methods

### Materials

SH-SY5Y cells were from Professor R. Ross (Fordham University, NY, USA). FluoroSave, fura-2/AM and thapsigargin were from Calbiochem (Darmstadt, Germany); all other chemicals were from Sigma-Aldrich (Dorset, UK) unless stated otherwise.

### Cell Culture and Establishment of N-Type and S-Type Predominant Populations

SH-SY5Y and S-type enriched populations were cultured in Dulbecco’s Modified Eagle Medium (DMEM)/F12:1 with GlutaMAX™ (Life Technologies, Paisley, UK), supplemented with foetal calf serum (10% *v*/*v*), penicillin (100 IU ml^−1^) and streptomycin (100 IU ml^−1^), and incubated at 37 °C in a humidified 5% CO_2_ atmosphere. Cells were passaged once a week with 0.02% ethylenediaminetetraacetic acid (EDTA) when approximately 90% confluent and were not used beyond passage 25. SH-SY5Y populations consist predominantly of N-type cells and were used in experiments to represent N-type predominant populations. S-type predominant populations were established by means of enrichment for substrate adherence through washing in PBS and gentle agitation to remove less adherent N-type cells (Bell 2013). S-type enrichment was repeated four times when cells were ~ 80% confluent.

### Differentiation and Cell Phenotype

Cells were seeded at least 24 h before treatment to allow sufficient cell adhesion and were subsequently treated with 1 μM 9*c*RA in complete media to induce differentiation or with 0.01% EtOH as a control treatment for proliferating cells. Differentiation and control media were replaced every 2 days for up to 7 days. After 7 days, treatment was withdrawn and cells were grown in complete media alone for a further 5 days. Proliferating and differentiating N-type predominant (P_N_ and D_N_ respectively) and S-type predominant (P_S_ and D_S_ respectively) populations were used for experiments from days 1 to 7 of treatment and after treatment withdrawal (WD). Proliferating N-type cells were identified on the basis of short, branched neurite-like processes arising from the small, rounded or slightly elongated cell bodies [6]. S-type cells had a flattened, spread morphology with no neurite-like processes, morphological criteria consistent (CLW, CPFR, TRC, unpublished data) with expression of the S-type markers vimentin and beta-tubulin III [6]. I-type cells were identified by a flattened morphology but with one or more neurite-like processes. Phase-contrast and differential interference contrast (DIC) images were used with MetaMorph software to measure cell differentiation after treatment. To measure differentiation of N-type cells, the total number of N-type cells and total number of differentiated cells, defined as possessing one or more neurites ≥ 50 μm in length, were counted manually. We have observed that S-type cells increase in diameter after 9*c*RA treatment and have used this as an objective criterion of differentiation in response to 9*c*RA.

### Immunofluorescence

Cells grown on glass coverslips were fixed with PFA (4% *w*/*v*), permeabilised using Triton X-100 (0.1% *v*/*v*) and blocked with bovine serum albumin (BSA, 5% *w*/*v*). Cells were incubated for 2 h at 4 °C with anti-STIM1 (1/50, BD Biosciences, USA) then incubated for 1 h at 4 °C with anti-mouse conjugate to FITC (Santa Cruz, USA). Coverslips were mounted onto glass slides with FluorSave Reagent. Images were captured using an Axiovert 200M confocal microscope (Carl Zeiss Ltd.) using a 63× oil immersion objective with 488-nm excitation light from an argon laser. Confocal images were acquired with the pinhole set at 1 Airy unit, 12-bit data depth, scan speed of 9 and mean of eight acquisitions. Images and quantification were obtained using LSM Image Browser (Zeiss) and ImageJ [23] software.

### Western Blotting

Cells were lysed in situ at 4 °C with lysis buffer containing 10% (*v*/*v*) Triton X-100, 2 mM Tris (pH 7.6), 1.28 mM sucrose, 1 mM EGTA (pH 8), 1 mM EDTA (pH 8) and protease inhibitor cocktail (Roche Diagnostics, Indianapolis, USA) then scraped from the plate and transferred to microcentrifuge tubes. Lysates were homogenised using a 20-G needle and centrifuged at 12,000 rpm for 10 min at 4 °C. Bradford assays were used to determine protein concentration in cell samples with BSA as a protein standard. The absorbance of samples with Protein Assay Dye Reagent Concentrate (Bio-Rad, Hertfordshire, UK) added was measured at 595 nm. Gel electrophoresis through NuPAGE 10% Bis-Tris gels (Invitrogen, California, USA) was used to separate total protein (20–40 μg) in sodium dodecyl sulphate (SDS) sample buffer (2% *w*/*v* SDS, 5% *v*/*v* 2-mercaptoethanol, 10% *w*/*v* glycerol and bromophenol blue in 60 mM Tris, pH 6.8) by reference to Precision Plus Protein Dual Color Standards (Bio-Rad). Separated proteins were transferred to a nitrocellulose membrane (Bio-Rad) and blocked for 1 h at RT (5% *w*/*v* milk, 0.02% *v*/*v* Triton X-100 in PBS). Blots were incubated with primary antibodies in incubation buffer (2.5% *w*/*v* milk in PBS) overnight at 4 °C. Primary antibodies included anti-β-actin (1/10,000, Abcam, UK), anti-Bcl-2 (1/200, Santa Cruz, USA), anti-STIM1 (1/200, BD Biosciences) and anti-Orai1 (1/200). Membranes were washed in a washing buffer (2.5% *w*/*v* milk, 0.2% *v*/*v* Triton X-100 in PBS) and incubated with horseradish peroxidase-conjugated mouse or rabbit secondary antibodies (1/5000, Dako, Denmark) for 1 h at RT. Enhanced chemiluminescence (1.25 mM luminol in 0.1 M Tris-HCL (pH 8.5), 0.09 mM p-coumaric acid and 0.09% *v*/*v* hydrogen peroxide) was used to detect immunoreactive bands on Hyperfilm™ (GE Healthcare, Amersham, Buckinghamshire, UK). Samples loaded on gels were loaded in a time-course order, alternating, proliferating and differentiating cell samples, and analysed with respect to treatment effects using densitometry of bands (ImageJ software [23]) normalised to β-actin expression as the loading control. Data consisted of three to six complete 7-day time-course replicates, with data for a subsequent washout condition (analysed at day 12) and additional data from separate experiments at day 7.

### CRAC Channel Inhibition

Cells were treated with varying concentrations (0.1–20 μM) of the CRAC channel inhibitor N-[4-[3,5-bis(trifluoromethyl)-1H-pyrazol-1-yl]phenyl]-4-methyl-1,2,3-thiadiazole-5-carboxamide (BTP2) and used for experiments after 24 h of treatment.

### Single-Cell Ca^2+^ Add-Back Experiments

Cells were washed with Krebs buffer (10 mM glucose, 4.2 mM NaHCO_3_, 1.2 mM MgSO_4_, 1.2 mM KH_2_PO_4_, 4.7 mM KCl, 118 mM NaCl, 2 mM CaCl_2_, 200 μM sulfinpyrazone, 10 mM HEPES, pH 7.4) and loaded with the Ca^2+^-sensitive fluorescent dye fura-2/AM (3 μM) for 45 min at RT. After loading, cells were incubated in Krebs buffer for a further 30 min to allow de-esterification of fura-2/AM. Cells were washed in Ca^2+^-free Krebs buffer and mounted into a coverslip holder (custom-made), producing a chamber into which Ca^2+^-free Krebs buffer was added. Ratiometric imaging was utilised through detection of fura-2 fluorescence at an excitation wavelength of 340 nm (300 ms exposure) and 380 nm (80 ms exposure) and an emission wavelength of 510 nm using a Nikon Eclipse TE300 microscope. Images were obtained with a charge-coupled device camera (Micromax, Sony Interline Chip, Princeton Instruments, Trenton, NJ) using a 20× objective.

After establishment of a steady baseline, 200 nM thapsigargin (TG) was added to induce ER Ca^2+^ store depletion followed by 2 mM CaCl_2_ to induce SOCE. Measurements of changes in fluorescence ratio (FR) in single cells were recorded with MetaFluor software (Universal Imaging, Marlow, UK) and used as a representation of [Ca^2+^]_i_. Data were recorded in Microsoft Excel for each region of interest, and individual single-cell recordings were assigned to a morphological phenotype (N-type, S-type or I-type). The area under the curve (AUC), peak height (PH), rate of rise and rate of decline for the initial 300 s from the peak height for TG and CaCl_2_ responses were calculated using VBA-coded functions in a Microsoft Excel template spread sheet custom built by Dr. Graham Scholefield. AUC and PH basal Ca^2+^ entry, determined by the addition of DMSO followed by CaCl_2_, were subtracted from all experimental data.

### Statistical Analysis

Data are presented as means ± S.E.M. Statistical analysis was carried out using R version 3.1.2 [24] and the packages ‘effects’, ‘PerformanceAnalytics’, ‘car’, ‘lme4’, ‘MASS’, ‘afex’, ‘doParallel’ and ‘phia’. SOCE ‘area under calcium-entry curve’ (AUC) data for single cells were analysed after subtraction of the mean background AUC for cells without prior thapsigargin treatment per experiment, per cell type and per population (and per time point for time-course data). Negative values were regarded as zero, and all background-subtracted data were transformed by adding 0.001 to avoid zeros. Box-cox transformation was used to normalise the data where appropriate, and quantile-quantile plots were used to assess data normalisation. Linear mixed-effect models with experiment and coverslip within experiment as random effects were used (package ‘lme4’) to test the effects of cell population (n-enriched or s-enriched), differentiation (9*c*RA treatment) and time, on the calcium release (thapsigargin treatment) and entry responses (SOCE) of single cells. Since lme4 does not explicitly provide main or interaction effect probabilities, these were estimated using the function ‘mixed’ in the package ‘afex’. Interaction contrasts were estimated using the package ‘phia’. For protein expression and cell morphology data, relationships between data points are indicated on graphs using first- or second-order linear functions (protein expression) and logistic growth curves (morphology; package ‘drc’). For linear models of differentiation, responses with time after 9*c*RA treatment and population enrichment type were included as additive effects or interactions on the basis of model selection guided by ANOVA and Akaike’s information criterion (AIC). For Western blot densitometry data, first- or second-order mixed-effects linear models, in which ‘experiment’ was included as a random effect, were used to test the significance of differentiation and time effects, as above. The level of significance is indicated in figures as *P* < 0.05*, *P* < 0.01** and *P* < 0.001***; ns means not significant.

## Results

Ca^2+^ store release and add-back experiments were performed on N-type-enriched and S-type-enriched SH-SY5Y populations to measure changes in SOCE activity of single cells in response to 9*c*RA-induced differentiation. The N-type-enriched and S-type-enriched populations both contained N-type, S-type and I-type cells; studying the Ca^2+^ signal changes with differentiation at a single-cell level allows characterisation of phenotype-specific Ca^2+^ responses and how these are affected by differentiation in different cellular contexts. N-type-enriched populations consisted primarily of N-type cells (66%) but with S-type (26%) and I-type cells (8%); S-type-enriched populations consisted mainly of S-type cells (81%) but also contained N-type (11%) and I-type cells (8%).

### N-Type- and S-Type-Enriched Populations Respond Differently to 9*c*RA Treatment and Withdrawal

N-type-enriched and S-type-enriched populations were studied over 7 days of 9*c*RA treatment and for a further 5 days after removal of 9*c*RA. Morphological differentiation of N-type cells, expressed as the percentage of N-type cells with at least one neurite > 50 μm in length [6, 25–27], increased over the course of treatment in differentiating (9*c*RA-treated) but not proliferating (control-treated) N-type-enriched populations (Fig. 1a–d). S-type cells exhibit a more flattened, spread-out morphology after 9*c*RA treatment [6] with an increase in diameter over the course of 9*c*RA treatment compared to proliferating S-type-enriched populations (Fig. 1g–j).

Expression of the anti-apoptotic protein Bcl-2 is up-regulated after differentiation of neuronal SH-SY5Y cells with retinoic acid [28] and is a useful biochemical marker of the differentiation response to 9*c*RA which distinguishes between N- and S-type cells [6]. There was a steady and significant increase in Bcl-2 expression with time of 9*c*RA treatment in N-type-enriched populations, although this was a slower response than the increase in morphological differentiation (Fig. 1e, f). Bcl-2 expression also increased, although to a lower extent, in proliferating cells after 6 days or more in culture (Fig. 1e). Conversely, for S-enriched populations, there was a small effect of 9*c*RA in decreasing Bcl-2 expression over time compared to proliferating control cells (Fig. 1k, l).

After 9*c*RA withdrawal, N-type cells reverted from a differentiating phenotype, with a reduction in morphological differentiation and Bcl-2 expression; conversely, S-type cells retained a differentiated morphology and low Bcl-2 expression (Fig. 1). Thus, N- and S-type cells respond to 9*c*RA but the differentiation phenotype is maintained in S-type cells but not in N-type cells, for the latter as shown both by morphology and Bcl-2 expression.

### Phenotype-Specific Ca^2+^ Store Release and Cell Population Effects Are Evident after 9*c*RA-Induced Differentiation

**Fig. 2:**
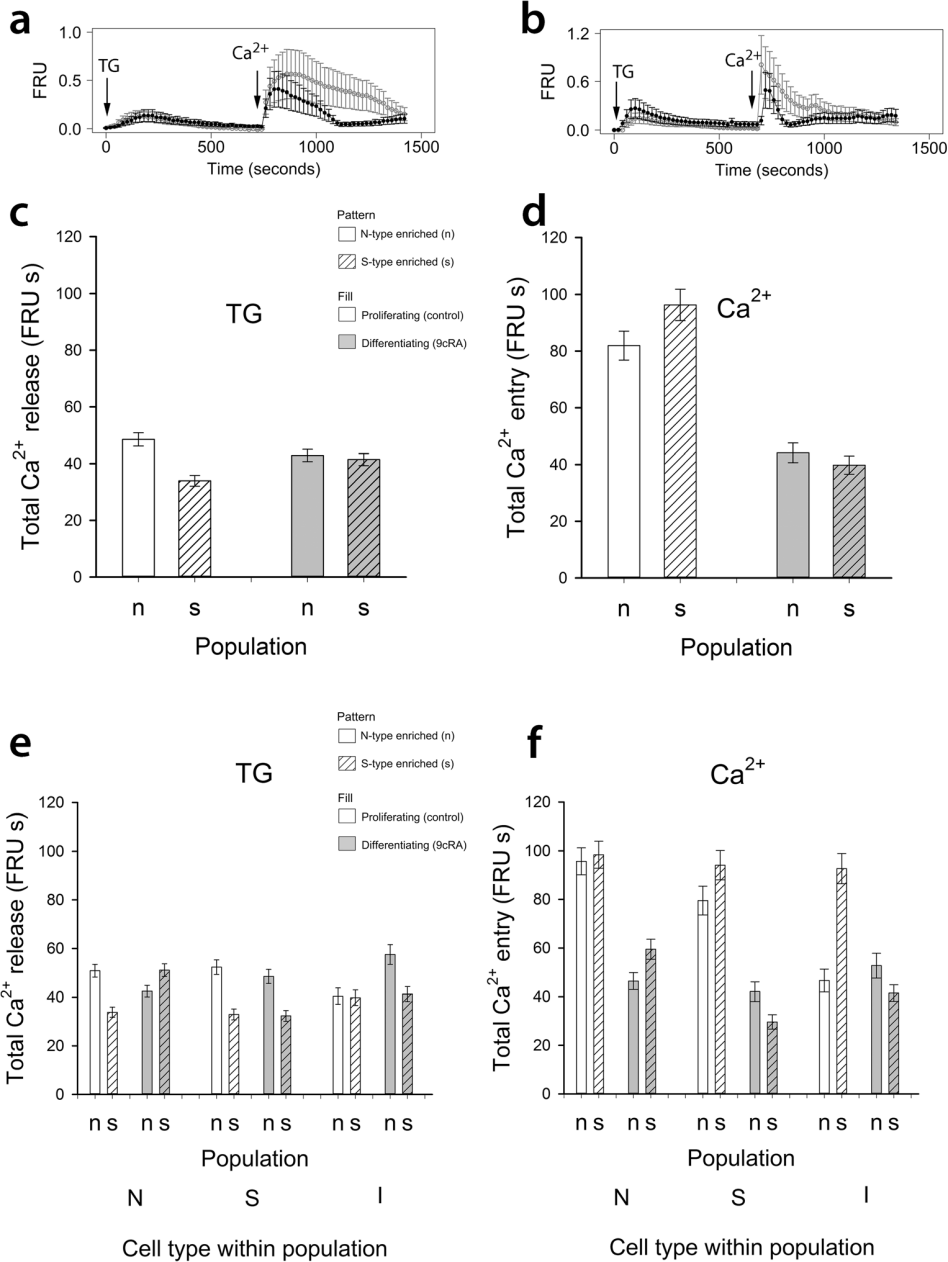
Uncoupling SOCE from ER Ca^2+^ store release in response to differentiation. In cells loaded with fura-2 and in Ca^2+^-free buffer, changes in fluorescence ratio units (FRU) reflect changes in [Ca^2+^]_i_ after adding thapsigargin (TG, 200 nM) to induce Ca^2+^ store release and subsequent re-addition of extracellular Ca^2+^ (CaCl_2_, 2 mM) to induce SOCE. Example traces from five cells combined (mean and SEM) are shown from N-type-enriched populations (‘n’) in **a**, and from S-type-enriched populations (‘s’) in **b**. Single-cell analysis of SOCE pathway activity (AUC; units FRU by time in seconds, abbreviated to FRU s) in ‘n’ and ‘s’ populations treated for 7 days with 1 μM 9*c*RA to induce differentiation or an equivalent volume of EtOH to maintain proliferation are shown in **c**–**f** (sample sizes in Table 2). With respect to Ca^2+^ release for cells within ‘n’ and ‘s’ populations overall (**c**), there was no significant effect of differentiation (*P* = 0.3) but population and interaction effects showed a significant decrease of Ca^2+^ release in cells in differentiating ‘n’ populations (*P* < 0.001) whereas Ca^2+^ release was increased in ‘s’ populations (*P* < 0.001). Comparisons of N-type cells within and between populations (**e**) showed that all main effects and interactions were significant: there was a significant decrease of Ca^2+^ release in N-type cells within ‘n’ populations but a significant increase of Ca^2+^ release in N-type cells within ‘s’ populations (*P* < 0.001). For S-type cells, there was no significant effect of differentiation (*P* = 0.16), or interaction, but a significant difference between populations (*P* < 0.001). All effects for I-type cells (population, differentiation) were significant (*P* < 0.001 for differentiation; *P* < 0.01 for population and interactions), but from the interaction effects, there was no difference in Ca^2+^ release as a result of differentiation of I-type cells within ‘s’ populations (*P* > 0.6) in contrast to the increased Ca^2+^ release after differentiation of I-type cells within ‘n’ populations (*P* < 0.001). For SOCE data for all cell types within populations (**d**), there was a significant effect of differentiation on reducing SOCE in both populations (*P* < 0.001), and this was also the case for N-type and S-type cells separately (**f**), although S-type cells had a bigger reduction of SOCE in ‘s’ populations (*P* < 0.001). For I-type cells, SOCE was significantly reduced by differentiation within ‘s’ populations (*P* < 0.001), a consequence of the high SOCE response of I-type cells in proliferating ‘s’ populations compared to proliferating ‘n’ populations. All error bars are ± SEM

Ca^2+^ store release in response to thapsigargin was examined in individual cells within N- and S-type-enriched populations, treated for 7 days with 9*c*RA or control (Fig. 2a, b). Analysis of single-cell data for all 5928 cells studied indicated no significant difference overall between proliferating and differentiating SH-SY5Y cells with respect to ER Ca^2+^ store release after addition of thapsigargin (*P* = 0.3, Fig. 2c). However, the effect of population type and the interaction with differentiation status was significant, due to opposite effects of 9*c*RA treatment on N-enriched and S-type-enriched populations, producing a decrease in the magnitude of Ca^2+^ store release in N-type-enriched populations but an increase in Ca^2+^ store release in S-enriched population cells (*P* < 0.001, Fig. 2c).

An examination of the responses of different cell phenotypes within the N- and S-type-enriched populations revealed cell type- and population-specific effects of differentiation on Ca^2+^ store release. For N-type cells, within N-enriched populations, 9*c*RA-induced differentiation produced a decrease in Ca^2+^ store release, but within S-enriched populations there was an increase (Fig. 2e), suggesting an effect of the predominant cell type on Ca^2+^ store release by N-type cells. Conversely, for S-type cells, 9*c*RA differentiation had no effect on Ca^2+^ store release (*P* = 0.16) either between or within populations. However, the level of the store release response differed between S-type cells in the N- and S-type-enriched populations (*P* < 0.001; Fig. 2e); S-type cells within N-enriched populations had greater Ca^2+^ store release compared to S-type cells within S-enriched populations, again indicating an effect of the predominant cell type on Ca^2+^ store release.

I-type cells had a different profile of Ca^2+^ store release response, depending on the enriched population. In N-enriched populations, I-type cells showed an increased Ca^2+^ store release (*P* < 0.001) after 9*c*RA treatment, but there was no effect of 9*c*RA-induced differentiation on I-type cells within S-enriched populations (*P* > 0.6; Fig. 2e).

### Total SOCE Is Altered with 9*c*RA-Induced Differentiation in a Cell Population and Phenotype-Specific Manner

After Ca^2+^ add-back, there was a significant decrease in SOCE after 9*c*RA-induced differentiation of both N- and S-type-enriched populations (*P* < 0.001; Fig. 2d). As with Ca^2+^ store release, there were population- and cell phenotype-specific differences. For N-type and S-type cells, regardless of enrichment population, 9*c*RA-induced differentiation produced a significant reduction in SOCE (*P* < 0.001, Fig. 2f). However, the magnitude of these effects differed between enriched populations, with a bigger effect of N-type cells within N-type enriched populations compared to within S-populations, and, conversely, for S-type cells the effect was bigger within S-enriched compared to N-enriched populations (*P* < 0.001, Fig. 2f). Again, this indicates an effect of population enrichment on the Ca^2+^ signalling properties of individual cell types. In marked contrast, there was no effect of 9*c*RA treatment on SOCE responses of I-type cells within N-enriched populations (*P* = 0.49), but a significant decrease in response to 9*c*RA within S-enriched populations (*P* < 0.001; Fig. 2f). Total SOCE was down-regulated by 53, 60 and 63% in N-type, S-type and I-type cells respectively, grown within differentiating N-enriched populations, and by 68, 68 and 61%, respectively, within S-enriched populations (Fig. 2f), and maximal SOCE was dampened after 9*c*RA-induced differentiation in all cell types within both population types (data not shown). These data demonstrate a marked uncoupling of SOCE from thapsigargin-mediated Ca^2+^ release from the ER in all cell types except I-type cells present in SH-SY5Y cell cultures enriched for N-type cells.

### SOCE Dynamics Are Altered with 9*c*RA-Induced Differentiation in a Population and Phenotype-Specific Manner

The rates of SOCE activation and initial SOCE deactivation were faster in proliferating and differentiating S-type-enriched populations compared to respective N-type-enriched populations (*P* < 0.001, Table 1); therefore, S-type enriched populations have a more transient SOCE response than N-type-enriched populations. For cells in both populations overall, the rate of SOCE activation increased after 9*c*RA differentiation (*P* < 0.05, Table 1). Individual N-type, S-type and I-type cells within S-enriched populations also had a more transient SOCE response as a result of higher rates of SOCE activation and initial deactivation compared to those cell types within N-enriched populations (*P* < 0.001, Table 2).

**Table 1:** Rates of SOCE activation and initial deactivation for cells in N-type- and S-type-enriched populations (± SEM)

Population	Status	SOCE activation rate (FRU s^−1^)	Initial SOCE deactivation rate (FRU s^−1^)
N-type enriched	Proliferating	49.4 ± 1.9	6.58 ± 0.181
N-type enriched	Differentiating	56.7 ± 1.7	7.37 ± 0.15
S-type enriched	Proliferating	87.0 ± 2.25	11.63 ± 0.212
S-type enriched	Differentiating	98.6 ± 3.54	9.66 ± 0.235

**Table 2:** Rates of SOCE activation and initial deactivation for individual cell types (N, S and I) in N-type- and S-type-enriched populations (± SEM). Sample sizes for N-type-enriched populations were 1204 proliferating (control) cells (761 N-, 307 S- and 136 I-type) and 1456 differentiating cells (1080 N-, 264 S- and 112 I-type). For S-type-enriched populations, there were 1897 proliferating (control) cells (679 N-, 1029 S- and 189 I-type) and 1371 differentiating cells (449 N-, 669 S- and 442 I-type)

Population	Status	SOCE activation rate (FRU s^−1^)	Initial SOCE deactivation rate (FRU s^−1^)
N-type cells			
N-type enriched	Proliferating	45.2 ± 1.8	6.0 ± 0.202
N-type enriched	Differentiating	54.0 ± 1.98	7.16 ± 0.167
S-type enriched	Proliferating	89.4 ± 4.14	12.2 ± 0.354
S-type enriched	Differentiating	116.3 ± 6.92	12.18 ± 0.445
S-type cells			
N-type enriched	Proliferating	59.3 ± 5.1	7.83 ± 0.394
N-type enriched	Differentiating	57.3 ± 3.37	6.95 ± 0.266
S-type enriched	Proliferating	86.25 ± 2.96	10.7 ± 0.27
S-type enriched	Differentiating	81.4 ± 4.29	7.54 ± 0.264
I-type cells			
N-type enriched	Proliferating	53.1 ± 9.08	7.56 ± 0.745
N-type enriched	Differentiating	79.8 ± 7.45	10.2 ± 0.798
S-type enriched	Proliferating	81.9 ± 5.05	14.4 ± 0.804
S-type enriched	Differentiating	109.04 ± 8.54	10.3 ± 0.586

The effects of differentiation with 9*c*RA on the dynamics of SOCE responses of different cell types varied between N- and S-enriched populations. For N-type cells, the SOCE response was more transient within N-enriched populations compared to N-type cells in S-enriched populations where the initial rates of deactivation did not increase after differentiation (Table 2). In contrast, for S-type cells within both populations, the SOCE response was less transient after differentiation, but the magnitude of this effect was bigger for S-type cells within S-enriched populations. Finally, for I-type cells the SOCE response was more transient in N-enriched populations after differentiation as a result of increases in rates of activation and initial deactivation, but within S-enriched populations, I-type cells had decreased initial rates of SOCE deactivation which may have counteracted the increased rates of activation in response to 9*c*RA (Table 2). Thus, there are population-specific and cell phenotype-specific effects of 9*c*RA on the dynamics of SOCE.

### Orai1 Is Down-Regulated and STIM1 Is Relocalised in Differentiating Cell Populations

**Fig. 3:**
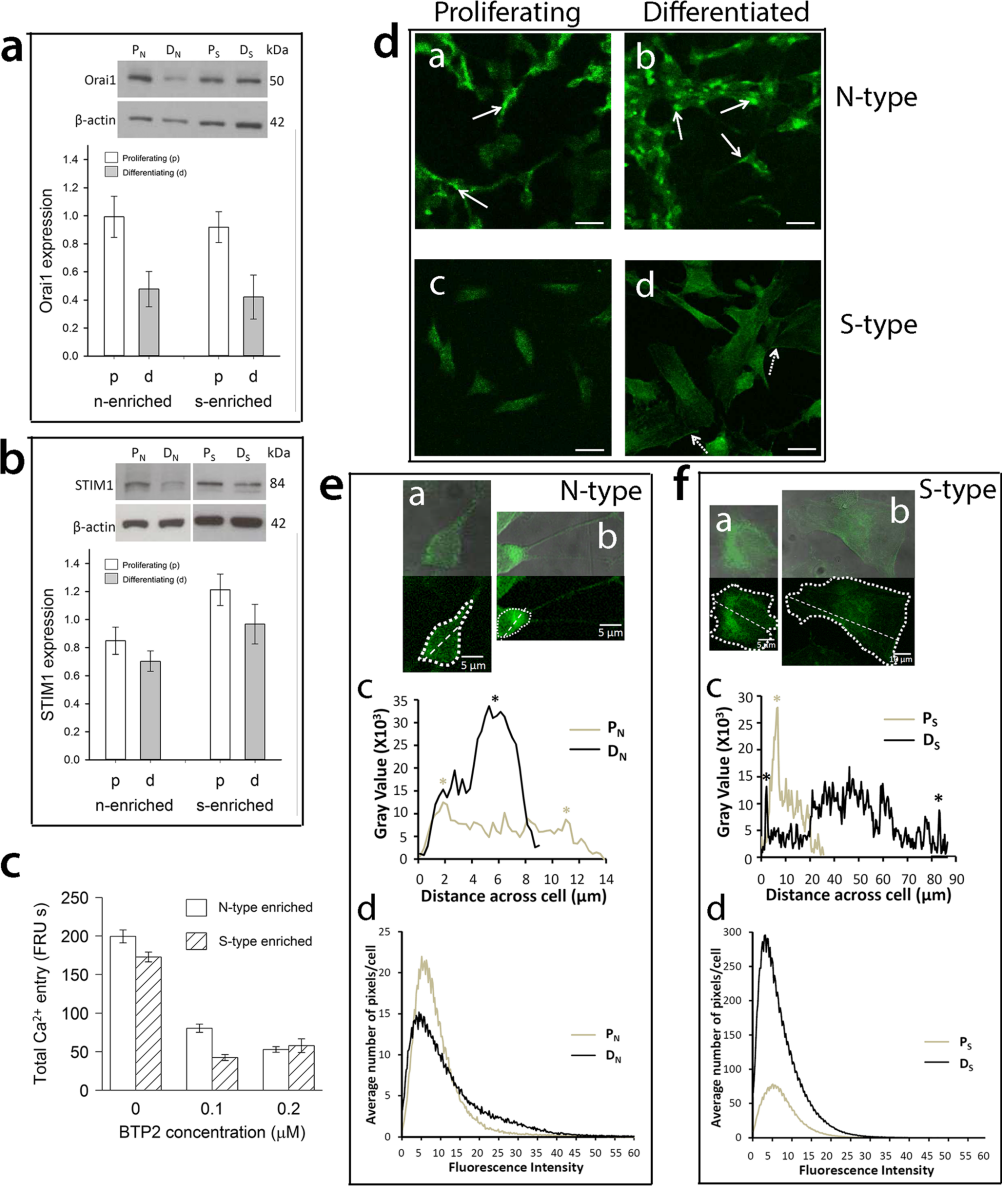
Altered expression of Orai1 and STIM1 and relocalisation of STIM1 in relation to 9*c*RA-induced differentiation of N-type- and S-type-enriched populations. N- (‘n’) and S-type-enriched (‘s’) populations were treated for 7 days with 1 μM 9*c*RA to induce differentiation (D_N_ and D_S_ respectively) or control vehicle to maintain proliferation (P_N_ and P_S_ respectively) and analysed for Orai1 (**a**) and STIM1 (**b**) expression by densitometry of Western blots using β-actin as a loading control. Orai1 expression, relative to β-actin, did not differ between populations (linear model ANOVA, *F*_1,29_ < 0.35, *P* > 0.5 for main effect and interaction) but was significantly reduced in expression by differentiation in both ‘n’ and ‘s’ populations (*F*_1,29_ = 13.1, *P* = 0.001). In contrast, STIM1 was expressed to a higher level overall in ‘s’ populations (ANOVA, *F*_1,58_ = 9, *P* < 0.01), but there was only a marginal effect of differentiation on both populations (differentiation *F*_1,58_ = 3.3, *P* = 0.075; interaction *F*_1,58_ = 0.23, *P* > 0.6). Cell populations were treated for 24 h with the CRAC-channel inhibitor BTP2 (**c**) at doses ranging from 0.1 to 20 μM and Ca^2+^ release in response to thapsigargin and SOCE analysed at a single-cell level within populations and between cell types within enriched populations. BTP2 at all doses (only 0.1 and 0.2 μM shown as no further reduction with increasing dose) did not affect thapsigargin-induced Ca^2+^ release but significantly reduced SOCE to similar extents in ‘n’ and ‘s’ populations (background corrected [no thapsigargin, by experiment, BTP2 dose, population and cell type]; ANOVA, effect of BTP2: *F*_1,3761/3463_ > 47, *P* < 0.001), with no differences between N-, S- and I-type cells within each population (*F*_2,3761_ = 2.1, *P* = 0.12, and *F*_2,3463_ = 0.92, *P* = 0.4 for ‘n’ and ‘s’ populations, respectively). Total Ca^2+^ entry shows AUC (units FRU by time in seconds, abbreviated to FRU s) of fluorescence traces after activation of SOCE by readdition of extracellular Ca^2+^. In P_N_ cells, STIM1 was present throughout the cytoplasm and neurite-like processes, with some evidence of localisation into foci (**d** (a) white arrows) whilst in D_N_ cells STIM1 formed distinct clusters (**d** (b) white arrows). In ‘s’ populations, STIM1 was present throughout P_S_ cells (**d** (c)) with no apparent clustering whilst in D_S_ cells there were clear examples of cells with an accumulation of STIM1 at the PM (**d** (d), dashed arrows). Scale bars represent 20 μm. The distribution of STIM1 was measured across the length of a proliferating and differentiated cell within an ‘n’ population (**e** (a) and **e** (b), respectively), and within an ‘s’ population (**f** (a) and **f** (b), respectively), as shown by dashed straight lines. The linear profile immunofluorescence pixel intensity over the distance of the cell bodies shown was quantified. STIM1 expression was distributed relatively evenly across P_N_ cells with evidence of some STIM1 foci in the cytoplasm (*) whereas in the cytoplasm of D_N_ cells STIM1 expression was less evenly distributed and instead formed distinct clusters (*) (**e** (c)). For ‘s’ populations, STIM1 expression was greatest in cytoplasmic regions close to the nucleus of P_S_ cells (*), and there was an accumulation of STIM1 at the membrane (*) and close to the nucleus of D_S_ cells (**f** (c)). Scale bars represent 5 μm (**e** (a, b), **f** (a)) or 10 μm (**f** (b)). There was an increased range of fluorescence intensities with highest pixel intensities in differentiating cells compared to proliferating cells (**e** (d) and **f** (d)). In ‘n’ populations, a shift in the number of low (P_N_) to high (D_N_) intensity pixels is consistent with differentiation-induced clustering of STIM1 (**e** (d)). Single-cell images (**e**, **f**) are representative of P_N_ *n* = 125, D_N_ = 125, P_S_ = 129, D_S_ = 128 from *N* ≥ 4, with ≥ 3 fields of view and ≥ 15 cells per experiment

Orai1 forms the PM CRAC channel pore and is required for cytosolic Ca^2+^ entry to replenish depleted ER Ca^2+^ stores [29–31]. Orai1 was expressed by proliferating and differentiating N-type-enriched and S-type-enriched populations (Fig. 3a) and, compared to proliferating populations, was significantly down-regulated by 52–54% in differentiating populations regardless of phenotype (ANOVA, *P* = 0.001, Fig. 3a), consistent with the extent of SOCE reduction (Fig. 2c). Furthermore, CRAC inhibition by BTP-2 in both N-type-enriched and S-type-enriched populations substantially suppressed SOCE activity (Fig. 3c) which is consistent with the role of Orai1 in SOCE within N-type-enriched and S-type-enriched populations [6].

**Fig. 4:**
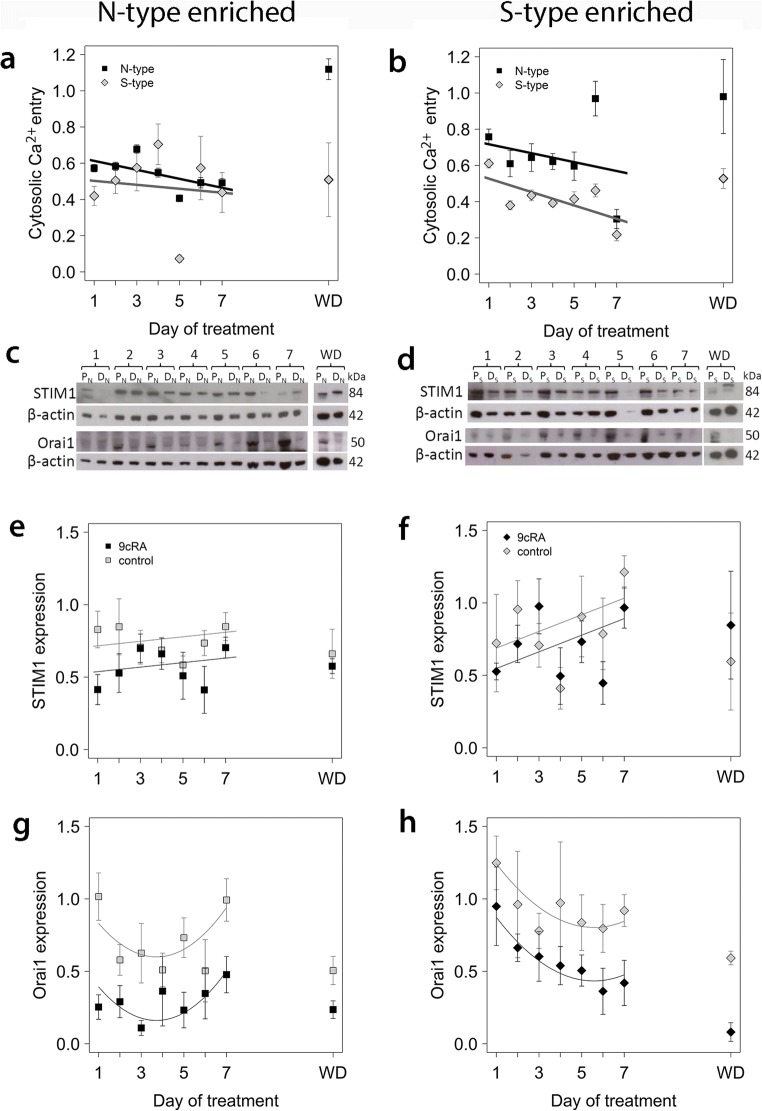
Time course of changes in SOCE in N-type-enriched (‘n’) and S-type-enriched (‘s’) populations in relation to differentiation and changes in expression of STIM1 and Orai1. N-type (left column) and S-type (right column) populations were treated for up to 7 days with 1 μM 9*c*RA (D_N_ and D_S_, respectively) to induce differentiation or control vehicle to maintain proliferation (P_N_ and P_S_, respectively); treatment was subsequently withdrawn and cells grown in standard culture medium for a further 5 days (WD). For the analysis of changes in SOCE at the single-cell level for N-type and S-type cells within ‘n’ (**a**) and ‘s’ (**b**) populations, all data were corrected for background calcium in the absence of thapsigargin. Data for SOCE of differentiating cells were expressed as a proportion of SOCE in proliferating cells at the relevant time point, normalised by Box-Cox transformation and analysed using linear mixed-effects models with experiment and coverslip within experiment as random effects. *P* values for ANOVA were obtained by simulation. SOCE was rapidly down-regulated by differentiation in both populations and cell types within populations and continued to decrease over 7 days (*P* < 0.001). For ‘n’ populations (**a**), there was no significant difference between N-type and S-type cells (*P* > 0.9), but for ‘s’ populations (**b**) reduction in SOCE was significantly lower for S-type cells than for N-type cells (*P* < 0.001). After washout in both population types, SOCE of N-type cells returned to values similar to proliferating cells, but SOCE of S-type cells remained depressed. Densitometry of Western blots (**c**, **d**) was used to examine the expression of STIM1 (**e**, **f**) and Orai1 (**g**, **h**) in ‘n’ (**c**, **e**, **g**) and ‘s’ (**d**, **f**, **h**) populations. For STIM1 expression in ‘n’ populations (**e**), there was a small but significant reduction in response to differentiation compared to proliferating cultures (first-order linear additive mixed-effects model, *P* < 0.001), but no significant change with time of culture (*P* = 0.8). Similarly, for ‘s’ populations, there was a small reduction of STIM1 expression in differentiated cells (*P* = 0.025), and no significant effect of time in culture (*P* = 0.9). For Orai1 expression, second-order linear mixed-effects models gave a better fit to the data, with differentiation significantly reducing Orai1 expression compared to proliferating cultures in both ‘n’ and ‘s’ populations (*P* < 0.001), a difference that was maintained after washout (**g**, **h**). In the case of ‘s’ populations, the data suggest a significant decline with time in culture (orthogonal polynomial linear term, *P* < 0.01 for ‘s’ populations; no effect of time in ‘n’ populations, *P* > 0.1). All error bars are ± SEM

The Ca^2+^-sensing protein STIM1 alters gating of PM CRAC channels through direct interaction with Orai1 to induce Ca^2+^ entry and replenish depleted ER Ca^2+^ stores [15, 18, 32]. STIM1 was expressed by N-type-enriched and S-type-enriched populations in proliferating and differentiating conditions, but to higher levels in S-type-enriched populations (ANOVA, *P* = 0.004; Fig. 3b). The apparent reduction of STIM1 expression in 9*c*RA-differentiated cells of both populations was not statistically significant, but of possible biological interest given the low *P* value (ANOVA, differentiation, *P* = 0.075; differentiation/population interaction, *P* = 0.63) and time-course data (Fig. 4).

There was evidence of a change in localisation of STIM1 after 9*c*RA-induced differentiation, and clear differences in localisation between N-type and S-type cells. In resting proliferating N-type cells, STIM1 was expressed uniformly with some evidence of foci (Fig. 3d, e). Upon differentiation, STIM1 expression became less evenly distributed and distinctly clustered (Fig. 3d), as quantified by measurements of linear profile immunofluorescence pixel intensity (Fig. 3e (a–c)). STIM1 was expressed throughout resting proliferating and differentiating S-type cells with an abundance of STIM1 close to the nucleus (Fig. 3d, f); however, there were clear examples of differentiating S-type cells with an accumulation of STIM1 at the PM (Fig. 3d, f). There was an increase in the range of STIM1 fluorescence intensity as well as higher fluorescence intensities in differentiating compared to proliferating N-type and S-type cells (Fig. 3e (d), f (d)), reflecting the accumulation of STIM1 into cytoplasmic clusters in differentiating N-type cells and at the PM of differentiating S-type cells.

### Remodelling of SOCE in Cell Populations Is Associated with Initiation and Maintenance of the Differentiated Phenotype

To understand the relationship between SOCE uncoupling and the differentiation response, we investigated the time course of changes in SOCE activity and SOCE protein expression after addition of 9*c*RA to N-type-enriched and S-type-enriched populations. SOCE was recorded in individual cells, and data, representing two to five experiments per day for 1–7 days of treatment with 9*c*RA, were analysed in relation to N-type and S-type cells within N-enriched and S-enriched populations; the number of I-type cells measured per time point was too small for meaningful analysis. Compared to proliferating N-type-enriched populations, within 24 h of 9*c*RA treatment, total SOCE was down-regulated by > 40% (Fig. 4a) in differentiating N-type-enriched populations, with an apparently greater response in S-type cells (*P* < 0.01; Fig. 4a) and a gradual decline over a 7-day period. This SOCE response coincided with a 50% down-regulation of STIM1 expression (Fig. 4c, e), a 75% down-regulation of Orai1 expression (Fig. 4c, g) and morphological differentiation (Fig. 1). These results suggest that 9*c*RA-induced differentiation of N-type-enriched populations induces an uncoupling of SOCE from ER Ca^2+^ store release within the first day of treatment. After treatment withdrawal for 5 days, total SOCE returned to that of proliferating cells (Fig. 4a), in accordance with the loss of biochemical differentiation (Fig. 1e, f). Whilst STIM1 levels were similar in proliferating and differentiating cells after treatment withdrawal, in the 9*c*RA-treated cells there was evidence from the Western blots of an increase in apparent molecular weight of STIM1 and Orai1 levels remained low (Fig. 4c, g).

In S-type-enriched populations, within 24 h of 9*c*RA treatment, there was also a substantial down-regulation of total SOCE, and to a greater extent in S-type cells compared to N-type cells (*P* < 0.01; Fig. 4b). STIM1 and Orai1 expression was also decreased in differentiating cells (Fig. 4d, f, h). The extent of SOCE activity down-regulation gradually increased with time of treatment (Fig. 4b). After treatment withdrawal, total SOCE in N-type cells within differentiated S-type-enriched populations recovered and was not significantly different from that of proliferating S-enriched populations (*P* > 0.05, Fig. 4b). Conversely, for S-type cells within S-type-enriched populations, SOCE remained depressed after 9*c*RA withdrawal compared to proliferating cells (Fig. 4b). Orai1 expression also remained down-regulated compared to proliferating populations (Fig. 4d, h). As with N-type-enriched populations, there was similar evidence of an increase in STIM1 molecular weight after 9*c*RA withdrawal (Fig. 4d). Overall, these data mirror the changes in cell morphology (Fig. 1) where S-type cells remained differentiated despite withdrawal of 9*c*RA, and suggest a link between SOCE responses and the differentiation status of N- and S-type cells.

**Fig. 5:**
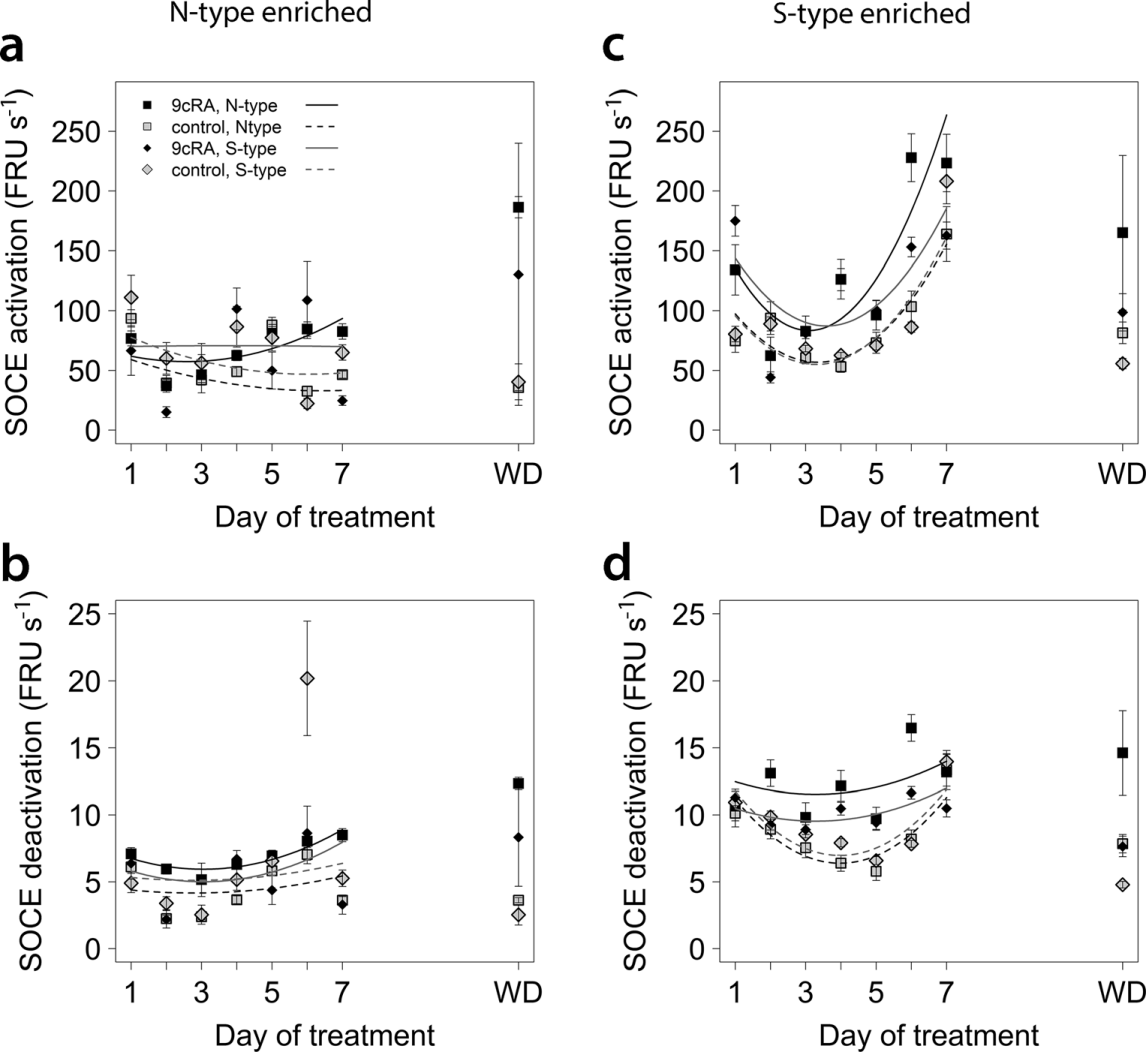
Changes in SOCE kinetics with time of treatment. Experimental material and details as in Fig. 4. For all graphs, the key is as shown in **a**. Fitted lines are linear second-order polynomial models of changes in SOCE kinetics with time for each cell type and differentiation status. Statistical comparisons were done using linear mixed effects models for the effects of time (day, second-order orthogonal polynomial) and interactions with cell type (N- or S-type) and differentiation status (differentiating or proliferating). For both populations, SOCE activation rates (**a**, ‘n’ population; **c**, ‘s’ populations) and initial deactivation rates (**b**, ‘n’ populations; **d**, ‘s’ populations) changed significantly with time and with differentiation (*P* < 0.01), but differentiation had greater effects on N-type cells regardless of population (*P* < 0.001) except that the difference in activation rates between N- and S-type cells in ‘s’ populations was marginal (*P* = 0.09). Overall, there were no significant differences in relation to cell type (*P* > 0.04), except that initial SOCE deactivation rates were lower for S-type cells in ‘s’ populations (*P* < 0.001). SOCE activation and initial deactivation rates remained elevated in differentiating N-type cells after washout regardless of population, and in differentiating S-type cells in ‘s’ populations, but in ‘s’ populations, SOCE activation and initial deactivation rates of differentiating S-type cells were more similar to proliferating cells

The changes in SOCE in response to differentiation were also accompanied by changes in SOCE kinetics (Fig. 5). For cells in N-type-enriched and S-type-enriched populations, 9*c*RA treatment increased SOCE activation and deactivation rates (*P* < 0.01), with differentiation having greater effects on N-type cells in both populations (*P* < 0.001), except for activation rates in S-enriched populations where the difference between N- and S-type cells was marginal, *P* = 0.09). Overall, both cell types responded in similar ways (*P* > 0.04), except that SOCE initial deactivation rates were lower for S-type cells in S-type-enriched populations (*P* < 0.001, Fig. 5d). After washout, SOCE activation and initial deactivation rates remained elevated in differentiating N-type cells regardless of population, and in differentiating S-type cells in N-type-enriched populations, whereas in S-type-enriched populations, SOCE activation and initial deactivation rates of differentiating S-type cells were more similar to proliferating cells (Fig. 5).

## Discussion

Previous findings from this laboratory have shown that SOCE becomes down-regulated in mixed [22] and enriched [6] populations of 9*c*RA-differentiated SH-SY5Y neuroblastoma cells. In this study, we have used single-cell analysis to characterise SOCE remodelling in differentiating N-type, S-type and I-type SH-SY5Y cells to determine how the remodelling response of the cell phenotypes influences that of the SH-SY5Y cell population as a whole. Our work reveals a surprising finding that Ca^2+^ signal remodelling in differentiating SH-SY5Y cells is independent of morphological phenotype but is apparently modulated by the cellular environment.

SH-SY5Y populations enriched for N-type or S-type cells both differentiated morphologically in response to 9*c*RA, but in a dissimilar manner with respect to cell shape, timing and Bcl-2 expression. In addition, Ca^2+^ signals were remodelled in both populations, but with different characteristics with respect to Ca^2+^ release and SOCE. Upon withdrawal of differentiating agent, N-type cells showed morphological and biochemical ‘de-differentiation’, whereas S-type cells retained a morphologically and biochemically differentiating phenotype. The cell types therefore differ in their phenotypic stability; N-type (neuronal) cells are phenotypically labile, whilst S-type (glial) cells are phenotypically stable.

In both cell populations, differentiation-induced Ca^2+^ signal remodelling included down-regulation of SOCE responses and Orai1 expression. Interestingly, the remodelled differentiating N-type phenotype was not fully restored when N-type populations were de-differentiated, since SOCE returned to pre-differentiation levels whilst Orai1 expression remained depressed. One possibility is that de-differentiation of N-type cells involves remodelling of the CRAC channel from Orai1 to Orai2 and/or Orai3 [33–35]. In support of this, de-differentiated N-type cells had higher rates of SOCE activation and initial SOCE deactivation (Fig. 5). This is consistent with studies showing that heterologously expressed Orai2 and Orai3 conduct CRAC-like currents with distinct biophysical characteristics from Orai1, including different activation and inactivation kinetics [34, 36].

STIM1 was also remodelled in a cell phenotype-dependent manner in differentiating SH-SY-5Y cells: both STIM1 expression and localisation were altered in N- and S-type cells. There was evidence of a slight increase in STIM1 molecular weight with 9*c*RA-induced differentiation of both phenotypes that was more apparent after 9*c*RA withdrawal. Given that this coincided with a relocalisation of STIM1 in both cell types, there may be a post-translational modification or shift in predominant isoform of STIM1 with differentiation. One possibility is up-regulation of the longer spliced variant, STIM1L [37, 38]. STIM1L has been proposed to form permanent clusters which allow the immediate activation of SOCE [37]. This would be consistent with the appearance of STIM1 clusters in differentiating N-type cells and the increased SOCE activation rates observed in differentiating cells.

In addition to the remodelling of SOCE, 9*c*RA-induced differentiation also resulted in the remodelling of TG-induced Ca^2+^ release in both cell types: in N-type populations, there was a decrease in Ca^2+^ store release whilst in S-type populations there was an increase. To investigate how remodelling of the population Ca^2+^ response is influenced by the predominant cell phenotype, we examined responses of different cell phenotypes within N- and S-type-enriched populations. For N-type cells within N-type-enriched populations, 9*c*RA treatment resulted in a decrease in TG-induced Ca^2+^ release. However, for N-type cells within S-type-enriched populations, 9*c*RA treatment resulted in an increase in Ca^2+^ store release. This suggests an effect of the predominant cell type on Ca^2+^ store release by N-type cells. Similarly, S-type cells within N-type-enriched populations had greater Ca^2+^ store release compared to S-type cells within S-type-enriched populations, again indicating an effect of the predominant cell type of Ca^2+^ store release. The reduction in Ca^2+^ store release after differentiation of N-type populations is entirely consistent with greater lability of the Ca^2+^ store reported previously in these cells [39], and this remodelling could contribute to the reduced stability of the differentiating N-phenotype compared with the differentiating S-phenotype reported here. For differentiating I-type cells also, the remodelled Ca^2+^ release response was dependent upon the enriched population. Interestingly, the remodelled SOCE response of differentiating I-type cells was dependent upon the enriched population, whereas the remodelled SOCE response of differentiating S-type and N-type cells was not (i.e. SOCE was down-regulated in differentiating S-type and N-type cells, irrespective of cellular context, whereas in I-type cells, SOCE was only down-regulated within S-type-enriched populations). This observation suggests that I-type cells have the greatest potential of all the cell phenotypes for remodelling their Ca^2+^ responses.

The SH-SY5Y neuroblastoma cell line has been used for many years as a model for elucidating signalling mechanisms in neurogenesis [3, 40] and disease [2, 41–44]. The finding that Ca^2+^ signalling is remodelled in both differentiating N-type and S-type cells, but with different characteristics with respect to Ca^2+^ release and SOCE, supports the idea that these phenotypes represent distinct lineages of neuronal (N-type) and glial (S-type) cells. Furthermore, the changes in biophysical characteristics of Ca^2+^ signals (e.g. extent of Ca^2+^ store release, SOCE activation and inactivation rates) that accompany differentiation concurs with evidence that different isoforms of STIM and Orai proteins predominate in neurons and glial cells [33, 35, 45]. This reinforces the idea that STIM and Orai isoforms could represent possible drug targets for the treatment of neuronal or glial dysfunction that may underlie neurodegenerative disorders.

In addition, the results of the present study also emphasise the importance of Ca^2+^ channels in cancer, particularly the regulation of interactions between different tumour components and implications for cell phenotype and tumorigenicity. The greater stability of the differentiating phenotype of S-type cells reported here concurs with the lower malignant potential of S-type neuroblastoma cells compared to N-type cells [46]. Critically, I-type cells may have the greatest malignant potential [47], and the greater potential of I-type cells to remodel their Ca^2+^ responses is a new dimension with substantial clinical and therapeutic implications. The different SOCE properties of I-type cells in N-type-enriched populations provide validation of the morphological criteria used as the basis of cell classification. However, although the properties of these I-type cells may reflect a characteristic of the N-type-enriched environment, these I-type cells may also be mechanically distinct from I-type cells in S-type-enriched populations; one possibility is that these are I-type cells detached easily by virtue of cell cycle stage and with SOCE properties influenced by their environment. Alternatively, I-type cells with different substrate-adherent properties may be more immediate progenitors of N-type and S-type cells, respectively, representing an intermediate phenotypic stage in conversion from I-type to N- or S-type, or may represent distinct lineage-restricted precursors to these phenotypes that have hitherto been unrecognised. Addressing these questions will demand different experimental approaches to elucidate the lineage and phenotypic characteristics of I-type cells.

This study also raises the question of the role of Ca^2+^ signalling mechanisms in neuroblastoma cell survival. We have previously shown that neuroblastoma cells respond to RA-induced differentiation by induction of an arachidonic acid (AA)-mediated survival mechanism [48]. Since these cells also show up-regulation of a non-SOCE and non-voltage-dependent Ca^2+^ entry pathway [22], one possibility is that an up-regulated arachidonate-regulated Ca^2+^ (ARC) entry pathway may have a role in cell survival. However, since Orai1 and STIM1 are involved in ARC as well as in SOCE [49], and the down-regulation of one of more of these components [50] or proteins that interact with them [51, 52] may protect tumour cells from apoptosis, dynamic modelling approaches are needed to clarify how such signalling processes interact in the context of cell differentiation and tumour biology.

The mechanism by which Ca^2+^ signal remodelling in differentiated cell phenotypes is modulated by the surrounding cellular environment is unknown. However, the finding is reminiscent of the bystander effect in cellular senescence [53] in that senescent cells spread senescence towards their neighbours by producing and secreting bioactive molecules [54, 55] or via gap junction-mediated cell-cell contact [53]. Although cellular senescence and terminal differentiation are distinct physiological process [56], it is noteworthy that both are forms of proliferative control that involve essentially permanent exit from the cell cycle [57]. A stress-induced bystander effect in neuronal stem progenitor cells induced by glioblastoma and medulloblastoma cells has been reported [58]; however, the present results raise the possibility that the effect is also seen in normal, unstressed neuronal cells. Enrichment of neuronal tumours with a specific cell type may affect the magnitude of the bystander effect that they may exert.

In summary, these results demonstrate the unique biology of neuronal and glial derivatives of common precursors and reveals the complexity of Ca^2+^ signal remodelling, particularly in relation to potential reciprocal interactions between survival via ARC/arachidonate-mediated Ca^2+^ signalling and SOCE in remodelled differentiated cells. This study also shows that direct or indirect interactions between cell types are important modulators of signalling processes in relation to neurogenesis. This requires detailed assessment in experimental models. Our results also highlight that the many studies using neuroblastoma derivatives as experimental models need to take account of how cellular heterogeneity could be influenced by culture techniques, as well as impact upon conclusions drawn from the tested population, and report these accordingly.

## References

[1] Kriegstein A, Alvarez-Buylla A (2009). The glial nature of embryonic and adult neural stem cells. Annu Rev Neurosci.

[2] Xicoy H, Wieringa B, Martens GJM (2017). The SH-SY5Y cell line in Parkinson’s disease research: a systematic review. Mol Neurodegener.

[3] Shipley MM, Mangold CA, Szpara ML (2016) Differentiation of the SH-SY5Y human neuroblastoma cell line. J Vis Exp 108: e53193. 10.3791/53193 10.3791/53193 PMC4828168 26967710

[4] Ciccarone V, Spengler BA, Meyers MB, Biedler JL, Ross RA (1989). Phenotypic diversification in human neuroblastoma cells—expression of distinct neural crest lineages. Cancer Res.

[5] Ross RA, Spengler BA, Biedler JL (1983). Coordinate morphological and biochemical interconversion of human neuroblastoma cells. J Natl Cancer Inst.

[6] Bell N, Hann V, Redfern CPF, Cheek TR (2013). Store-operated Ca2+ entry in proliferating and retinoic acid-differentiated N- and S-type neuroblastoma cells. Biochim Biophys Acta.

[7] Ross RA, Biedler JL (1985). Presence and regulation of tyrosinase activity in human neuro-blastoma cell variants in vitro. Cancer Res.

[8] Berridge MJ, Bootman MD, Lipp P (1998). Calcium—a life and death signal. Nature.

[9] Bootman MD, Lipp P, Berridge MJ (2001). The organisation and functions of local Ca2+ signals. J Cell Sci.

[10] Sammels E, Parys JB, Missiaen L, De Smedt H, Bultynck G (2010). Intracellular Ca2+ storage in health and disease: a dynamic equilibrium. Cell Calcium.

[11] Smyth JT, Hwang SY, Tomita T, DeHaven WI, Mercer JC, Putney JW (2010). Activation and regulation of store-operated calcium entry. J Cell Mol Med.

[12] Luik RM, Wang B, Prakriya M, Wu MM, Lewis RS (2008). Oligomerization of STIM1 couples ER calcium depletion to CRAC channel activation. Nature.

[13] Stathopulos PB, Zheng L, Ikura M (2009). Stromal interaction molecule (STIM) 1 and STIM2 calcium sensing regions exhibit distinct unfolding and oligomerization kinetics. J Biol Chem.

[14] Zhang SYL, Yu Y, Roos J, Kozak JA, Deerinck TJ, Ellisman MH, Stauderman KA, Cahalan MD (2005). STIM1 is a Ca2+ sensor that activates CRAC channels and migrates from the Ca2+ store to the plasma membrane. Nature.

[15] Kawasaki T, Lange I, Feske S (2009). A minimal regulatory domain in the C terminus of STIM1 binds to and activates ORAI1 CRAC channels. Biochem Biophys Res Commun.

[16] Liou J, Kim ML, Heo WD, Jones JT, Myers JW, Ferrell JE, Meyer T (2005). STIM is a Ca2+ sensor essential for Ca2+−store-depletion-triggered Ca2+ influx. Curr Biol.

[17] Wu MM, Buchanan J, Luik RM, Lewis RS (2006). Ca2+ store depletion causes STIM1 to accumulate in ER regions closely associated with the plasma membrane. J Cell Biol.

[18] Park CY, Hoover PJ, Mullins FM, Bachhawat P, Covington ED, Raunser S, Walz T, Garcia KC, Dolmetsch RE, Lewis RS (2009). STIM1 clusters and activates CRAC channels via direct binding of a cytosolic domain to Orai1. Cell.

[19] Parekh AB, Putney JW (2005). Store-operated calcium channels. Physiol Rev.

[20] Putney JW (1986). Identification of cellular activation mechanisms associated with salivary secretion. Annu Rev Physiol.

[21] Putney JW (2011). The physiological function of store-operated calcium entry. Neurochem Res.

[22] Brown AM, Riddoch FC, Robson A, Redfern CPF, Cheek TR (2005). Mechanistic and functional changes in Ca2+ entry after retinoic acid-induced differentiation of neuroblastoma cells. Biochem J.

[23] Schneider CA, Rasband WS, Eliceiri KW (2012). NIH image to ImageJ: 25 years of image analysis. Nat Methods.

[24] R Core Team (2016). R: a language and environment for statistical computing.

[25] Dwane S, Durack E, Kiely PA (2013). Optimising parameters for the differentiation of SH-SY5Y cells to study cell adhesion and cell migration. BMC Res Notes.

[26] Lovat PE, Lowis SP, Pearson ADJ, Malcolm AJ, Redfern CPF (1994). Concentration-dependent effects of 9-cis retinoic acid on neuroblastoma-differentiation and proliferation in-vitro. Neurosci Lett.

[27] Takenobu H, Shimozato O, Nakamura T, Ochiai H, Yamaguchi Y, Ohira M, Nakagawara A, Kamijo T (2011). CD133 suppresses neuroblastoma cell differentiation via signal pathway modification. Oncogene.

[28] Lasorella A, Iavarone A, Israel MA (1995). Differentiation of neuroblastoma enhances Bcl-2 expression and induces alterations of apoptosis and drug resistance. Cancer Res.

[29] Demuro A, Penna A, Safrina O, Yeromin AV, Amcheslavsky A, Cahalan MD, Parker I (2011). Subunit stoichiometry of human Orai1 and Orai3 channels in closed and open states. Proc Natl Acad Sci U S A.

[30] Hoover PJ, Lewis RS (2011). Stoichiometric requirements for trapping and gating of Ca2+ release-activated Ca2+ (CRAC) channels by stromal interaction molecule 1 (STIM1). Proc Natl Acad Sci U S A.

[31] Mignen O, Thompson JL, Shuttleworth TJ (2008). Orai1 subunit stoichiometry of the mammalian CRAC channel pore. J Physiol Lond.

[32] Sauc S, Bulla M, Nunes P, Orci L, Marchetti A, Antigny F, Bernheim L, Cosson P, Frieden M, Demaurex N (2015). STIM1L traps and gates Orai1 channels without remodeling the cortical ER. J Cell Sci.

[33] Kraft R (2015). STIM and ORAI proteins in the nervous system. Channels.

[34] Lis A, Peinelt C, Beck A, Parvez S, Monteilh-Zoller M, Fleig A, Penner R (2007). CRACM1, CRACM2, and CRACM3 are store-operated Ca2+ channels with distinct functional properties. Curr Biol.

[35] Moccia F, Zuccolo E, Soda T, Tanzi F, Guerra G, Mapelli L, Lodola F, D'Angelo E (2015). Stim and Orai proteins in neuronal Ca2+ signaling and excitability. Front Cell Neurosci.

[36] DeHaven WI, Smyth JT, Boyles RR, Putney JW (2007). Calcium inhibition and calcium potentiation of Orai1, Orai2, and Orai3 calcium release-activated calcium channels. J Biol Chem.

[37] Darbellay B, Arnaudeau S, Bader CR, Konig S, Bernheim L (2011). STIM1L is a new actin-binding splice variant involved in fast repetitive Ca2+ release. J Cell Biol.

[38] Horinouchi T, Higashi T, Higa T, Terada K, Mai Y, Aoyagi H, Hatate C, Nepal P, Horiguchi M, Harada T, Miwa S (2012). Different binding property of STIM1 and its novel splice variant STIM1L to Orai1, TRPC3, and TRPC6 channels. Biochem Biophys Res Commun.

[39] Riddoch FC, Brown AM, Rowbotham SE, Redfern CPF, Cheek TR (2007). Changes in functional properties of the caffeine-sensitive Ca2+ store during differentiation of human SH-SY5Y neuroblastoma cells. Cell Calcium.

[40] Kovalevich J, Langford D (2013). Considerations for the use of SH-SY5Y neuroblastoma cells in neurobiology. Methods Mol Biol.

[41] Agholme L, Lindstrom T, Kagedal K, Marcusson J, Hallbeck M (2010). An in vitro model for neuroscience: differentiation of SH-SY5Y cells into cells with morphological and biochemical characteristics of mature neurons. J Alzheimers Dis.

[42] Lopes FM, Schroder R, da Frota ML, Zanotto-Filho A, Muller CB, Pires AS, Meurer RT, Colpo GD, Gelain DP, Kapczinski F, Moreira JC, Fernandes Mda C, Klamt F (2010). Comparison between proliferative and neuron-like SH-SY5Y cells as an in vitro model for Parkinson disease studies. Brain Res.

[43] Petratos S, Li QX, George AJ, Hou X, Kerr ML, Unabia SE, Hatzinisiriou I, Maksel D, Aguilar MI, Small DH (2008). The beta-amyloid protein of Alzheimer’s disease increases neuronal CRMP-2 phosphorylation by a Rho-GTP mechanism. Brain.

[44] Schneider L, Giordano S, Zelickson BR, M SJ GAB, Ouyang X, Fineberg N, Darley-Usmar VM, Zhang J (2011). Differentiation of SH-SY5Y cells to a neuronal phenotype changes cellular bioenergetics and the response to oxidative stress. Free Radic Biol Med.

[45] Verkhratsky A, Parpura V (2014). Store-operated calcium entry in neuroglia. Neurosci Bull.

[46] Spengler BA, Lazarova DL, Ross RA, Biedler JL (1997). Cell lineage and differentiation state are primary determinants of MYCN gene expression and malignant potential in human neuroblastoma cells. Oncol Res.

[47] Walton JD, Kattan DR, Thomas SK, Spengler BA, Guo HF, Biedler JL, Cheung NKV, Ross RA (2004). Characteristics of stem cells from human neuroblastoma cell lines and in tumors. Neoplasia.

[48] Bell E, Ponthan F, Whitworth C, Westermann F, Thomas H, Redfern CPF (2013). Cell survival signalling through PPAR delta and arachidonic acid metabolites in neuroblastoma. PLoS One.

[49] Shuttleworth TJ (2012). STIM and Orai proteins and the non-capacitative ARC channels. Front Biosci.

[50] Flourakis M, Lehen'kyi V, Beck B, Raphael M, Vandenberghe M, Vanden Abeele F, Roudbaraki M, Lepage G, Mauroy B, Romanin C, Shuba Y, Skryma R, Prevarskaya N (2010). Orai1 contributes to the establishment of an apoptosis-resistant phenotype in prostate cancer cells. Cell Death Dis.

[51] Albarran L, Lopez JJ, Woodard GE, Salido GM, Rosado JA (2016). Store-operated Ca2+ entry-associated regulatory factor (SARAF) plays an important role in the regulation of arachidonate-regulated Ca2+ (ARC) channels. J Biol Chem.

[52] Jardin I, Albarran L, Salido GM, Lopez JJ, Sage SO, Rosado JA (2017). Fine-tuning of store-operated calcium entry by fast and slow Ca(2+)-dependent inactivation: involvement of SARAF. Biochim Biophys Acta.

[53] Nelson G, Wordsworth J, Wang C, Jurk D, Lawless C, Martin-Ruiz C, von Zglinicki T (2012). A senescent cell bystander effect: senescence-induced senescence. Aging Cell.

[54] Coppe JP, Patil CK, Rodier F, Sun Y, Munoz DP, Goldstein J, Nelson PS, Desprez PY, Campisi J (2008). Senescence-associated secretory phenotypes reveal cell-nonautonomous functions of oncogenic RAS and the p53 tumor suppressor. PLoS Biol.

[55] Passos JF, Nelson G, Wang C, Richter T, Simillion C, Proctor CJ, Miwa S, Olijslagers S, Hallinan J, Wipat A, Saretzki G, Rudolph KL, Kirkwood TB, von Zglinicki T (2010). Feedback between p21 and reactive oxygen production is necessary for cell senescence. Mol Syst Biol.

[56] Buttitta LA, Edgar BA (2007). Mechanisms controlling cell cycle exit upon terminal differentiation. Curr Opin Cell Biol.

[57] Talluri S, Isaac CE, Ahmad M, Henley SA, Francis SM, Martens AL, Bremner R, Dick FA (2010). A G1 checkpoint mediated by the retinoblastoma protein that is dispensable in terminal differentiation but essential for senescence. Mol Cell Biol.

[58] Sharma N, Colangelo NW, de Toledo SM, Azzam EI (2016). Diffusible factors secreted by glioblastoma and medulloblastoma cells induce oxidative stress in bystander neural stem progenitors. ASN Neuro.

